# Intravenous versus Epidural Routes of Patient-Controlled Analgesia in Abdominal Surgery: Systematic Review with Meta-Analysis

**DOI:** 10.3390/jcm11092579

**Published:** 2022-05-05

**Authors:** Dmitriy Viderman, Karina Tapinova, Fatima Nabidollayeva, Ramil Tankacheev, Yerkin G. Abdildin

**Affiliations:** 1 Department of Biomedical Sciences, Nazarbayev University School of Medicine (NUSOM), Kerei, Zhanibek khandar Str. 5/1, Nur-Sultan 020000, Kazakhstan; karina.tapinova@nu.edu.kz; 2 Department of Anesthesiology and Intensive Care, National Research Oncology Center, Kerei, Zhanibek khandar Str. 3, Nur-Sultan 020000, Kazakhstan; 3 Department of Mechanical and Aerospace Engineering, School of Engineering and Digital Sciences, Nazarbayev University, 53 Kabanbay Batyr Ave., Nur-Sultan 010000, Kazakhstan; fatima.nabidollaeyeva@nu.edu.kz (F.N.); yerkin.abdildin@nu.edu.kz (Y.G.A.); 4 Pain Management Department, National Neurosurgery Center, 34/1 Turan Ave., Nur-Sultan 010000, Kazakhstan; ramil.tankacheev@nmh.kz

**Keywords:** patient-controlled analgesia, epidural analgesia, intravenous analgesia, pain control, abdominal surgery, postoperative pain

## Abstract

Objective: To compare the intravenous and epidural routes of patient-controlled anesthesia in abdominal surgery. Methods: We searched for randomized clinical trials that compared the intravenous and epidural modes of patient-controlled anesthesia in intra-abdominal surgery in adults. Data analysis was performed in RevMan 5.4. Heterogeneity was measured using I^2^ statistic. Risk of bias was assessed using the Jadad/Oxford quality scoring system. Results: Seven studies reporting 529 patients were included into the meta-analysis. For pain at rest, the mean difference with 95% confidence interval (CI) was −0.00 [−0.79, 0.78], *p*-value 0.99, while for pain on coughing, it was 0.43 [−0.02, 0.88], *p*-value 0.06, indicating that patient-controlled epidural analgesia (PCEA) was superior. For the sedation score, the mean difference with 95% CI was 0.26 [−0.37, 0.89], *p*-value 0.42, slightly favoring PCEA. For the length of hospital stay, the mean difference with 95% CI was 1.13 [0.29, 1.98], *p*-value 0.009, favoring PCEA. For postoperative complications, the risk ratio with 95% CI was 0.8 [0.62, 1.03], *p*-value 0.08, slightly favoring patient-controlled intravenous analgesia (PCIVA). A significant effect was observed for hypotension, favoring PCIVA. Conclusions: Patient-controlled intravenous analgesia compared with patient-controlled epidural analgesia was associated with fewer episodes of hypotension. PCEA, on other hand, was associated with a shorter length of hospital stay. Pain control and other side effects did not differ significantly. Only three studies out of seven had an acceptable methodological quality. Thus, these conclusions should be taken with caution.

## 1. Introduction

Abdominal surgery is a frequent and definitive management option for a wide variety of abdominal diseases. Postoperative pain is one of the major issues that are frequently not appropriately controlled. Inadequate perioperative pain management is associated with nausea, vomiting, ileus, delayed ambulation, prolonged hospital stays, and increased total cost of hospitalization [1]. Patient-controlled analgesia (PCA) is considered the gold standard in postoperative pain management after major abdominal surgery, providing better results compared to standard intravenous opioid analgesia [2,3]. Existing patient-controlled analgesia techniques allow patients to titrate analgesics in small doses to optimize pain control and minimize the side effects [2,3,4]. Delivery of opioids via patient-controlled intravenous analgesia (PCIVA) improves pain relief compared to nurse administration and requires less nursing, while the risks of opioid-related side effects, such as respiratory depression, sedation, nausea, and vomiting, are similar [4,5,6]. Previous studies have confirmed that PCIVA is preferred over nurse-administered opioids [4]. Continuous intravenous infusion of opioids compared with PCIVA results in a postoperative period associated with a significant increase in the incidence of respiratory depression [7]. Patient-controlled epidural anesthesia (PCEA) is another option of analgesic administration controlled by the patient. PCEA can employ opioids, local anesthetics, or both [8]. PCEA is believed to reduce the surgery-related sympathetic activity via a reduction of stimulation of the central nervous system. Other benefits of epidural patient-controlled analgesia include early gastrointestinal recovery after surgery [8]. These benefits are believed to be more visible in patients with a high risk of postoperative pulmonary and cardiac complications [9]. The benefit of these “patient-controlled” methods is that patients can control their own analgesia through an electronic controller. Whenever more analgesia is needed, patients can administer a predetermined dose of analgesic solution and titrate opioids depending on the individual pain intensity. Several previous randomized controlled trials demonstrated that thoracic epidural analgesia with opioids and local anesthetics resulted in a reduction of pain intensity compared with PCA at rest and on coughing [10,11,12]. Furthermore, multiple studies showed benefits of epidural use, such as a reduction in the rates of systemic side effects attributed to opioids delivered through PCIVA, e.g., sedation and respiratory depression and bowel dysfunction. Epidural PCA contributes to early ambulation and early return to normal activities [10,11,12]. Conversely, epidural analgesia is invasive, time-consuming, requires technical skills, is more expensive [13], and carries risks of serious complications, such as infection, nerve injury, and paralysis [8]. Moreover, the rates of analgesic failure or malfunctioning of epidural catheters can be high [4].

The purpose of this meta-analysis was to compare PCIVA and PCEA in intra-abdominal surgery.

## 2. Materials and Methods

This meta-analysis was conducted and is reported according to the Preferred Reporting Items for Systematic Reviews and Meta-Analyses (PRISMA) guidelines.

**Figure 1:**
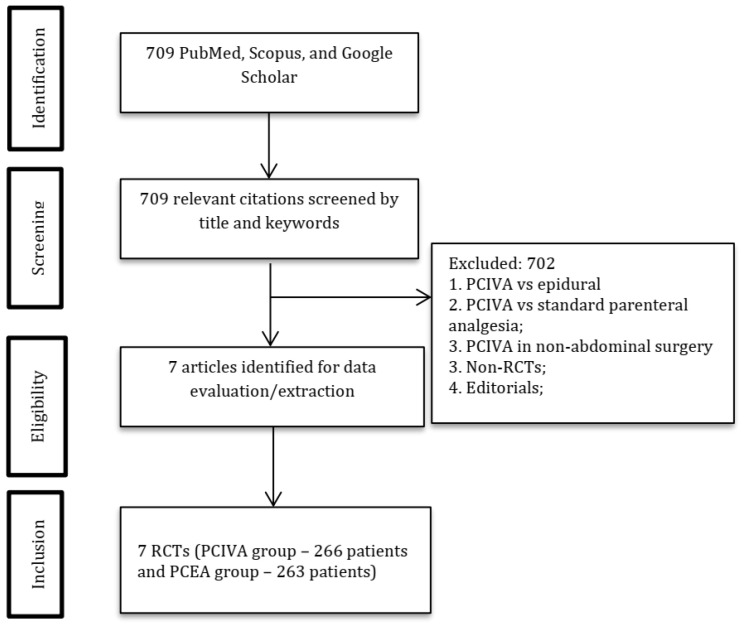
PRISMA diagram. Abbreviations: PCIVA, patient-controlled intravenous analgesia; PCEA, patient-controlled epidural analgesia.

One author (DV) searched for relevant articles in PubMed, Google Scholar, and the Cochrane Library published before October 2021 (Figure 1).

The following search terms or their combination “patient-controlled analgesia”, “patient-controlled intravenous analgesia”, “intravenous patient-controlled analgesia”, “patient-controlled epidural analgesia”, “epidural patient-controlled analgesia”, “abdominal surgery” were used during the search. The following keyword combinations were used for PubMed: (((((patient-controlled analgesia) OR (patient-controlled intravenous analgesia)) OR (intravenous patient-controlled analgesia)) OR (patient-controlled epidural analgesia)) OR (epidural patient-controlled analgesia)) AND (abdominal surgery).

### 2.1. Criteria for Including Studies

Types of studies: we considered only randomized controlled trials (RCTs).

Age of participants: 18 years and older.

Types of surgical procedures: open surgeries, liver, gastric, intestinal, urologic, and gynecologic surgeries.

Timing of outcomes: the outcomes were evaluated at any time during the period of the individual studies.

### 2.2. Exclusion Criteria

Types of studies: non-RCTs, Editorials.

Types of participants: pediatric, under 18.

Types of surgical procedures: non-abdominal surgeries, laparoscopic surgeries.

The studies were checked for the correctness of the groups. Studies not reporting PCIVA vs. PCEA in abdominal surgery were excluded.

### 2.3. Primary Outcomes

Pain intensity score at rest and on movement (or when coughing) measured 24 h after surgery and assessed using a visual analogue scale (VAS) or a numeric rating scale (NRS), from 0 to 10 or from 0 to 100.

### 2.4. Secondary Outcomes

1. Side effects
  1. Respiratory depression (respiratory rate <10 per minute, hypoxemia (SpO_2_ < 90% by pulse oximetry), administration of opioid antagonists).
  2. Nausea and vomiting
  3. Pruritus
  4. Sedation
  5. Respiratory complications such as respiratory depression (respiratory rate less than 10 breaths per minute or requirement for an opioid antagonist), hypoxemia (defined as SpO_2_ < 90% by pulse oximetry).
  6. Hypotension leading to worsening conditions or requiring fluid or vasopressor administration.
2. Length of hospital stay (LoS) and length of stay in post-anesthesia care unit or intensive care unit (if reported).

### 2.5. Assessment of Methodological Quality and Critical Appraisal of Individual Studies

Two reviewers, DV and YA, appraised the quality of each study, independently. Any discrepancies were resolved by DV and YA by discussion until reaching a consensus or, if this was not possible, by inviting a third reviewer (KT). The methodological quality of the included studies was assessed using the Oxford quality scoring system (Jadad Scale) [14]. The quality of the studies was graded from 1 (min) to 5 (max) as low (<3), acceptable (3), good (4), and excellent (5).

### 2.6. Data Extraction and Statistical Methods

KT and FN independently collected data from published original articles. The data were rechecked by DV and YA. We entered the data into a data table. The following rubrics were included: reference, 1st author, year of publication, country, design and goals of the study, age of the participants, type of surgery, sample size, American Society of Anesthesiologists (ASA) physical status, pharmacological agents and adjuvants, and side effects.

The risk of bias due to missing results was addressed as follows. If studies had missing data values (e.g., sample standard deviations), we tried to estimate them by known estimation techniques using other statistics (e.g., 1st, 2nd, 3rd quartiles, 95% CIs). We calculated the sample mean and the sample standard deviation from data presented in the 1st quartile, median, 3rd quartile, and sample size using the methods developed by Luo et al. [15] for sample mean and by Wan et al. [16] for sample standard deviation. If such statistics were not reported, the study was not included into our meta-analysis.

Data analysis was conducted using the “Review Manager software (RevMan, version 5.4)”. It applies the Inverse Variance method for continuous data and the Mantel–Haenszel statistical method of analysis for dichotomous data by default and provides 95% CIs for each outcome. Pooled continuous outcomes were reported as mean difference with 95% confidence intervals, and pooled dichotomous outcomes were reported as risk ratio with 95% confidence intervals. Heterogeneity was assessed using I^2^ statistic. The sensitivity analysis was conducted by eliminating one study at a time to analyze the possible change of the results. Due to different populations examined in the studies, we used a random-effects meta-analysis in our study.

## 3. Results

The systematic search yielded 709 articles, of which 702 articles were excluded after screening. Seven articles reporting 529 patients (PCIVA group, 266 and PCEA group, 263) were selected for the meta-analysis [10,17,18,19,20,21,22] (Figure 1, Table 1). 

### 3.1. Postoperative Pain Scores at Rest (at 24 h)

**Figure 2:**
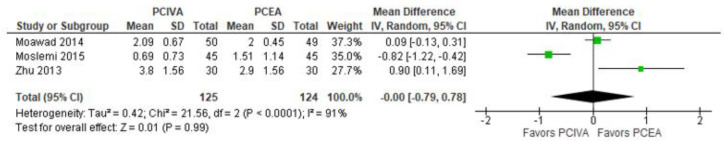
Postoperative pain scores at rest (at 24 h) [17,18,19].

Three studies reported the postoperative pain scores at rest [17,18,19]. The forest plot in Figure 2 shows no difference between PCEA and PCIVA: the mean difference with 95% CI was −0.00 [−0.79, 0.78]. The model showed high heterogeneity (I^2^ = 91%), which was significant (*p*-value = 0.0001). We utilized the mean difference since all studies used the same 0-10 scale. The total number of patients in the two groups was very close, i.e., 124 for PCEA, and 125 for PCIVA.

### 3.2. Postoperative Pain Scores on Coughing (at 24 h)

**Figure 3:**
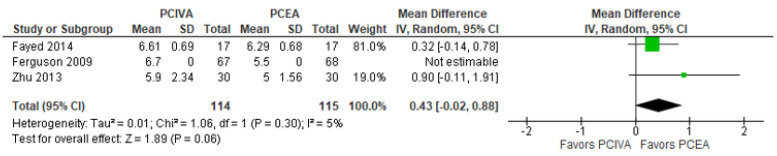
Postoperative pain scores on coughing (at 24 h) [19,20,21].

The model (Figure 3) showed no significant difference between PCEA and PCIVA [19,20,21]: the mean difference with 95% confidence interval (CI) was 0.43 [−0.02, 0.88]. The total number of patients in the PCIVA group was 114, and that in the PCEA group was 115. One study [21] did not provide the sample standard deviation, but the sample mean for the PCEA group was lower than the one for the PCIVA group, which supported the model’s overall result. The mean pain scores were higher for the PCIVA groups (6.61; 6.7; 5.9) than for the PCEA groups (6.29; 5.5; 5); therefore, the mean difference (PCIVA−PCEA) was positive, favoring PCEA, since a lower pain score is preferred.

### 3.3. Postoperative Sedation Score (at 24 h)

**Figure 4:**
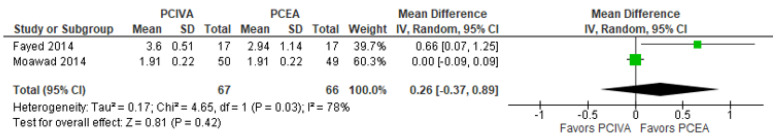
Postoperative sedation score (at 24 h) [17,20].

Only two studies [17,20] reported the postoperative sedation score. The model (Figure 4) did not show a clear advantage of PCEA over PCIVA: the mean difference with 95% CI was 0.26 [−0.37, 0.89]. The model showed high heterogeneity (I^2^ = 78%), which was significant (*p*-value = 0.03). The total number of patients in the two groups was very close, i.e., 66 in the PCEA group, and 67 in the PCIVA group.

### 3.4. Postoperative Complications

**Figure 5:**
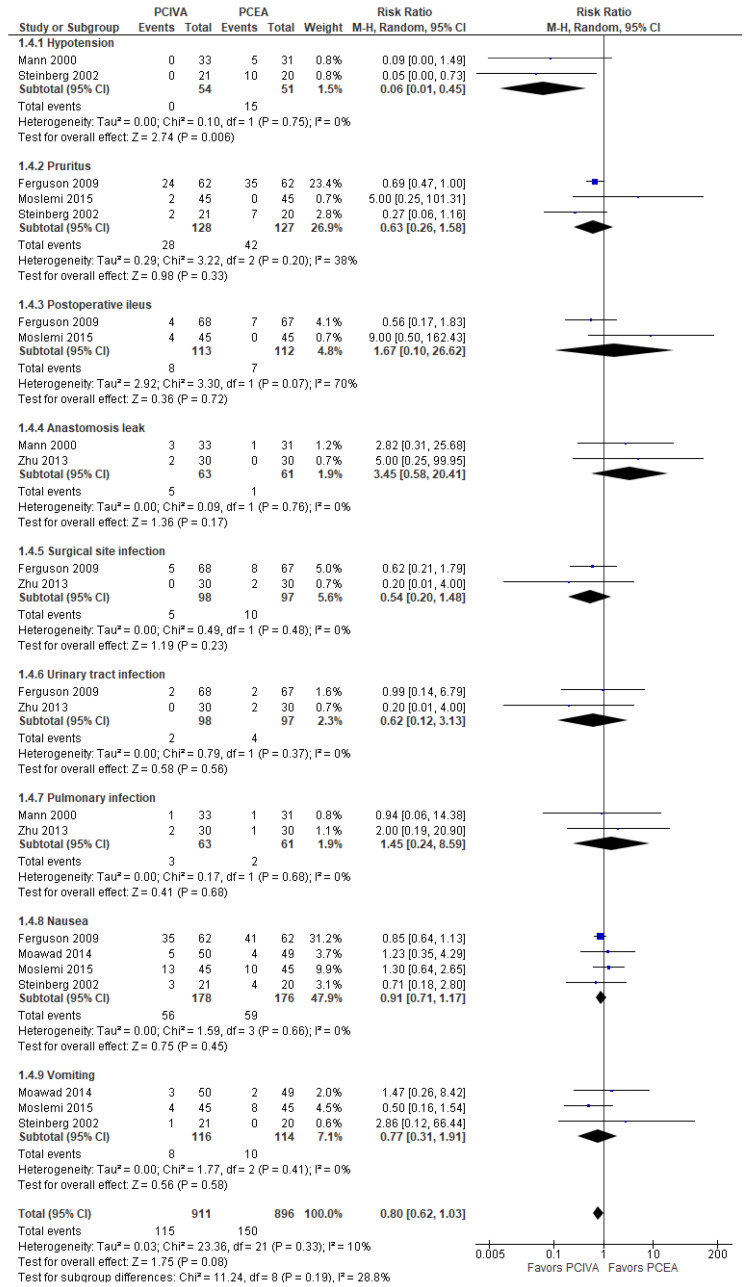
Postoperative complications [10,17,18,19,21,22].

Considering its overall effect, the model (Figure 5) did not favor PCIVA over PCEA; the risk ratio with 95% CI was 0.80 [0.62, 1.03], *p*-value = 0.08.

The subgroup analysis showed that the model favored PCIVA over PCEA only for hypotension; no patient suffered from hypotension in the PCIVA groups. The model did not show a significant difference of PCIVA over PCEA for other side effects, i.e., pruritus, postoperative ileus, anastomosis leak, surgical site infection, urinary tract infection, pulmonary infection, nausea, and vomiting.

### 3.5. Length of Stay (Days)

**Figure 6:**
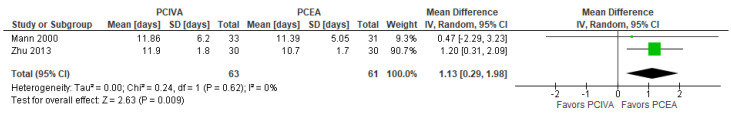
Length of hospital stay [10,19].

Regarding the length of hospital stay, the overall results of the model (Figure 6) indicated that PCEA was better than PCVIA; the mean difference with 95% CI was 1.13 [0.29, 1.98], *p*-value = 0.009.

### 3.6. Assessment of the Methodological Quality (Jadad/Oxford Quality Scoring System)

The methodological quality of four studies was graded as low, and that of three studies as acceptable. The grading of the included studies is presented in Table 2.

## 4. Discussion

This meta-analysis evaluated the effect of PCIVA and PCEA in intra-abdominal surgery. There was a small number of patients after the pooling the selected studies—no more than 125 and 124 participants in the PCIVA and PCEA groups, respectively. Three studies out of seven had an acceptable methodological quality, while four studies were graded as of low methodological quality. All the studies did not succeed in blinding, due to the different nature of the PCIVA and PCEA procedures. The selected trials included major open gynecologic cancer surgery, laparoscopic radical major gastric cancer surgery, hepatic resection, open colon surgery, and cephalic pancreatectomy.

For our primary objective, we compared pain at rest and on coughing within 24 h. Selected RCTs used 10-grade scales (VAS or NPRS). After pooling the results of all RCTs, there was no significant reduction in total pain intensity at rest. The model of pain on coughing favored PCEA but did not reach statistical significance. Rescue analgesia could affect these values. However, the trials did not provide comparable total opioid consumption for these patients. The mode of analgesia delivery was also different across the studies, which could cause high heterogeneity of the model of pain at rest (Table 1).

The implementation of PCEA was mainly based on establishing a more targeted (regional) drug delivery (mainly of opioids and local anesthetics) in the epidural space, limiting the systemic side effects of analgesics, achieving protection against surgical stimulation, and achieving additional beneficial effects through parasympathetic activation (early return of intestinal motility). Many of these effects have been well established by previous preclinical and clinical studies [4,5,6,7,8,9,10,11,12,23]. However, not all these positive effects of epidural anesthesia/analgesia are supported by the results of this MA. Some studies included in this MA reported that PCEA was superior in pain score reduction [16,19]; however, we failed to find statistical support for these findings. Moreover, it appears that some conclusions in the original articles were not sufficiently based on statistical analysis.

To date, there is insufficient evidence to conclude that PCEA is significantly better than PCIVA in postoperative pain control. In terms of safety, there is insufficient evidence supporting the effect of PCEA in the reduction of respiratory depression; the level of sedation was lower in the PCEA group, and the PCIVA group presented fewer episodes of hypotension.

Although the comparison of their analgesic efficacy is important, the decision whether to use PCIVA or PCEA should also be based on the individual characteristics of a particular patient, the risk of side effects, and contraindications.

One of the most significant limitations of this MA is the heterogeneity in terms of the association of PCIVA with fewer episodes of hypotension, the structure and reporting style of the published articles, and the types and anatomical location of the performed surgeries (e.g., major open gynecologic cancer surgery, major gastric cancer, prostatectomy, hepatic resection, open colon surgery, colectomy, and cephalic pancreatectomy). Some articles reported data in such a way that they could not be used in a meta-analysis (e.g., missing confidence intervals). Another limitation is a low number of matched clinical trials. The lack of statistical significance may be due to the insufficient number of articles included in the analysis. Finally, our literature search may not have been able to find all publications related to the review objectives.

To improve the quality of future RCTs, we might recommend the consistent use of standard outcomes, e.g., pain intensity at rest, pain intensity on movement or coughing, side effects, that is, systemic side effects of the drugs used for PCA, either IV or epidural—such as respiratory depression, sedation, nausea, vomiting, intestinal hypomotility/recovery of the gastrointestinal tract, itching, local anesthetic systemic toxicity—and side effects and complications of PCEA (mechanical complications, paralysis, hypotension). In the era of evidence synthesis, it becomes important that the number of original articles on such a research topic increases; unfortunately, some studies could not be included in the quantitative synthesis due to inconsistencies in the analysis and presentation of the results.

## 5. Conclusions

This meta-analysis demonstrated that PCIVA was associated with fewer episodes of hypotension compared to PCEA. Pain scores at rest or on coughing, postoperative sedation scores, shivering, delirium, respiratory depression, urinary tract infections, pulmonary infections, surgical site infections, nausea and vomiting did not differ significantly. All studies could not properly blind observers and participants due to the nature of the analgesia mode, and this significantly decreased the methodological quality of most of the studies. Three studies out of seven had an acceptable methodological quality, while the others had a low methodological quality. Overall, the pooled sample was small. Additional randomized controlled trials comparing these two patient-controlled methods are required to answer the question about their benefits and risks. Future randomized controlled trials should be of sufficient power to demonstrate the most clinically important outcomes, such as pain scores, side effects, recovery rate, and use a more standardized assessment and reporting format that would be more suitable for a quantitative synthesis.

## Figures and Tables

**Table 1:** Characteristics of the studies included in the meta-analysis.

Author, Citation	Country	Study Design	Study Goals	Age	N of Patients: Total (Intervention/Control)	Group	Diagnosis	Surgery	General Anesthesia	ASA	Dose of Opioids and Local Anesthetics	Postoperative Analgesia	Conclusions
Ferguson, 2009 [21]	USA	RCT	Primary—pain at rest and cough (VAS, 0–10). Secondary—GI and GU function, time to discharge	PCEA: 57 C: 55	135 (67/68)	PCEA C: IV PCA	Gynecologic cancer	Open GYN cancer surgery	Yes	-	PCEA: morphine 100 μg/mL, 0.05% bupivacaine, 4 mL/h. Rescue: 4 mL every 0.5 h	All: 15–30 mg IV ketorolac every 8 h, 2 post-op days PCEA: morphine 100 μg/mL, 0.05% bupivacaine, 4 mL/h, continuous Rescue: 4 mL every 0.5 h C: morphine, 1 mg/h, continuous Rescue PCA: 1 mg every 10 min	PCEA provides better analgesia after open GYN cancer resections
Zhu, 2013 [19]	China	RCT	Pain (VAS, 0–10), blood sugar, time to flatus, safety, duration of hospital stay	20-75 PCEA: 61.1 (12.6) PCIA: 59.6 (13.0)	60 (30/30)	PCEA: T8-T9 PCIA	Gastric cancer	Gastrectomy	Yes	-	PCEA: 0.05% bupivacaine, 100 μg/mL morphine, 4 mL/h, 2 days. Rescue: 4 mL, 0.5 h lockout	All: pethidine. PCEA: 0.05 % bupivacaine, 100 μg/mL morphine, 4 mL/h continuous, 2 days. Rescue: 4 mL, 0.5 h lockout PCIA: morphine, 1 mg/h, continuous. Rescue: 1 mg every 10 min	After gastrectomy, PCEA provided safer and better pain control and faster recovery of GI function.
Moawad, 2014 [17]	Egypt	RCT	Pain (NPRS, 0–10), rescue analgesia use, sedation, and patient satisfaction. Complications (PONV, shivering, pruritus, or pulmonary problems)	20-60 PCEA: 44.45 (10.56)PCIA: 45.20 (10.61)	99 (49/50)	PCEA: T10-12 PCIA: IV	-	Abdominal surgery	Yes	I, II	PCEA: bupivacaine 0.125%, fentanyl	All: Rescue 0.5 μg/kg IV fentanyl PCEA: fentanyl 5 μg/mL, bupivacaine 0.125% (1.25 mg/mL), bolus PCIA: fentanyl 20 μg/mL, bolus	PCEA demonstrated superior analgesia, less sedation, and higher patient satisfaction.
Moslemi, 2015 [18]	Iran	RCT	Primary—pain (VAS, 1–10). Secondary—rescue analgesia use, side effects (nausea, vomiting, ileus, pruritis, sedation, pulmonary problems)	40-60 IVPCA: 53.8 (11.4) PCEA: 49.9 (8.8)	90 (45/45)	PCEA: L2-L3 IVPCA	Ovarian, endometrial, cervical cancer	GYN cancer surgeries	Yes	I, II	PCEA: 0.5% bupivacaine 120 mg (24 mL) (bupivacaine hydrochloride 50 mg/20 mL), fentanyl 150 µg (3 mL) in saline. 6–8 mL/h, 2 mL every 15 min on demand.	All: rescue—pethidine (0.5 mg/kg IV) PCEA: 0.5% bupivacaine 120 mg (24 mL) (bupivacaine hydrochloride 50 mg/20 mL), fentanyl 150 µg (3 mL) in saline. 6–8 mL/h, 2 mL bolus every 15 min on demand IVPCA: 300 µg (6 mL) fentanyl, 200 mg (4 mL) pethidine, 8 mg (2 mL) ondansetron in 0.9% saline, total 100 mL. 6–8 mL/h, 2 mL bolus every 15 min, on demand.	Epidural analgesia provided lower sedation and less respiratory depression
Fayed, 2014 [20]	Egypt	RCT	Primary—pain. Secondary—side effects (sedation, PONV, urinary retention, pulmonary and neurological complications), recovery of GI function, duration of ICU and hospital stay.	P: 50.1 (9.7)E: 50.8 (11.5)	34 (17/17)	E: epidural T11-T12 P: IV PCA	Liver cirrhosis	Hepatic resection	Yes	I, II	E: bupivacaine 0.125%, 2 µg/mL fentanyl	All: IV pethidine, paracetamol E: bupivacaine 0.125%, 2 µg/mL fentanyl. 6 mL/h, continuous, 3 mL bolus every 15 min. P: IV fentanyl 15 μg, 10 min lockout, 90 μg/h maximum.	IV PCA and epidural analgesia are similarly efficient. Higher risk of coagulopathy in cirrhotic patients favors IVPCA.
Steinberg, 2002 [22]	USA	RCT	Primary—duration of hospital stay. Secondary—recovery, safety	18-80 PCEA: 62 (10) IV PCA: 61 (15)	41 (20/21)	PCEA IV PCA	-	Open colon surgery	Yes	I-III	PCEA: ropivacaine 0.2%, fentanyl (2 g/mL), 8 mL/h intraoperatively	All: ketorolac 15 mg. After 3 days, ibuprofen 400 mg PO 4/day until discharge or post-op day 6 PCEA: ropivacaine 0.2%, fentanyl (2 g/mL), 4 mL/h 2 mL ropivacaine/fentanyl bolus, 15 min lockout. Rescue—5 mL ropivacaine 2 mg/mL, fentanyl 2 g/mL, in 15 min. If needed, another bolus in 0.5 h. If inadequate analgesia—4–6 mL ropivacaine 7.5 mg/mL. IV PCA: morphine 0.1 mg/kg intraoperatively	PCEA offers better pain management, lowers opioid use, and enables faster recovery.
Mann, 2000 [10]	France	RCT	Primary—pain and side effects. Secondary—mental status and complications (GI, pulmonary, and hemodynamic)	PCA: 76.8 (4.7) PCEA: 76.1 (5.6)	70 (35/35)	IVPCA PCEA: T7-T9 or T9-T11	-	Colectomy, gastrectomy, cephalic panreatectomy	Yes	I, II	PCEA: intraoperative 0.25% bupivacaine, 1 pg/mL sufentanil, postoperative 0.125% bupivacaine, 0.5-pg/mL sufentanil, 2–3 mL, 12 min lockout, 3–5 mL/h	IV PCA: 1.5 mg morphine bolus, 8 min lockout. PCEA: 2–3 mL, 12 min lockout, 3–5 mL/h, continuous.	Epidural analgesia provides superior pain control and better mental and GI activity but did not improve postoperative delirium or complications rate.

Abbreviations: RCT—randomized controlled trials, PCEA—patient-controlled epidural analgesia, PCA—patient-controlled analgesia, GYN—gynecology, PCIA—patient-controlled intravenous analgesia, NPRS—Numeric Pain Rating Scale, PONV—postoperative nausea and vomiting, VAS—visual analogue scale, GI—gastrointestinal.

**Table 2:** Jadad scale.

Study or Subgroup	Was This Study Described as Randomized?	Was the Method Used to Generate the Sequence of Randomization Appropriate and Described?	Was the Study Described as Double-Blind?	Was the Method of Double-Blind Appropriate and Described?	Was There a Description of Withdraw and Dropouts?	Total Score
Fayed, 2014 [20]	1	0	0	0	0	1
Ferguson, 2009 [21]	1	0	0	0	1	2
Mann, 2000 [10]	1	1	0	0	1	3
Moawad, 2014 [17]	1	1	0	0	1	3
Moslemi, 2015 [18]	1	1	0	0	1	3
Steinberg, 2002 [22]	1	0	0	0	1	2
Zhu, 2013 [19]	1	0	0	0	1	2

## Data Availability

Not applicable.
